# An Investigation of Microstructural Evolution, Tensile Properties and Work-Hardening Behavior of Nanosized TiB_2_/Al-Cu-Mn Composites

**DOI:** 10.3390/ma13194250

**Published:** 2020-09-24

**Authors:** Cun Juan Xia, Lei Wang, Qing Zhang, Hao Fei Zhu, Jun Liu, Feng Guo Zhang, Zhe Chen, Hao Wei Wang

**Affiliations:** 1 State Key Laboratory of Metal Matrix Composites, Shanghai Jiao Tong University, Shanghai 200240, China; xiacunjuan@sjtu.edu.cn (C.J.X.); zhangqing0904@sjtu.edu.cn (Q.Z.); zhuhaofei183@sjtu.edu.cn (H.F.Z.); fg.zhang@sjtu.edu.cn (F.G.Z.); hwwang@sjtu.edu.cn (H.W.W.); 2 School of Materials Science and Engineering, Shanghai Jiao Tong University, Shanghai 200240, China; mseliujun@sjtu.edu.cn

**Keywords:** Al matrix composites, nanosized TiB_2_, microstructure evolution, tensile properties, work-hardening

## Abstract

The microstructure evolution, tensile properties and work-hardening behavior of AA2219 alloy reinforced by in situ nanosized TiB_2_ particles were studied in this paper. The observation indicated an impeded recrystallization of the matrix alloy by nanosized TiB_2_ particles, and the hybrids of nanosized TiB_2_ particles and Al_2_Cu phases located at the grain boundary hindered the grain growth. Meanwhile, a large amount fiber textures of <111>//RD (Rolling direction), <110>//RD, <100>//RD <111>//ND (Normal direction), <110>//ND and <100>//ND were detected in nanosized TiB_2_/AA2219 composite. Tensile test results exhibited a combination of good strength and ductility of the present composite whose yield strength and tensile strength were 11.4% and 5.8% higher than those of the alloy, while its fracture strain increased slightly. Meanwhile, the correlation between this modified microstructure of nanosized TiB_2_ particles and comprehensive mechanical properties was established. This study provides a new insight into the fabrication and strengthening behaviors of Al matrix composites reinforced by in situ nanoparticles.

## 1. Introduction

The specific strength and modulus are commonly considered two key parameters when assessing materials used in structural design and other practical applications. A good match of high mechanical performance (e.g., strength, ductility, elastic modulus) and physical properties (e.g., density) is thus necessary. To meet such demands, nanosized-particle-reinforced Al matrix composites (NPRAMCs) have been the promising materials used in practice [1,2,3,4,5,6], which consequently calls for the study of the influences of the reinforcement microstructure and matrix characteristics on mechanical behavior.

The published literature reveals enhancement in wear resistance, damping response, creep resistance and mechanical strength, etc., when nanoparticles are incorporated into Al matrix [7,8,9]. These nanometric particles can impede dislocation motion and accumulate dislocation inside grain interiors via the Orowan mechanism, resulting in a desired strengthening effect [10,11]. A notable discovery of strength–ductility synergy is achieved since the nanoparticles is lower than 1 vol.%, owing to the load transfer capacity [9,12]. For example, nanosized TiC_p_/Al-Cu composites prepared by Zhou et al. [13] showed high tensile strength and excellent elongation (11.08% and 187.9% higher than those of Al-Cu alloy). Liu et al. [14] utilized in situ techniques to prepare oxide nanoparticles in the interiors of Mo grains, acquiring a yield strength greater than 800 MPa and a tensile elongation of 40. However, a small number of reinforced particles limit the strengthening efficiency, while a larger portion of nanoparticles are difficult to incorporate uniformly into ductile matrix due to van der Waals attractive forces [15,16]. Those nanoparticle agglomerates will lead to increase in stress localization and thereby deteriorate the nanoparticle strengthening effect. Although great efforts have been made, preventing nanoparticles from agglomerating during processing remains a major challenge [16,17,18,19,20,21,22].

In addition, load transfer across a particle–matrix interface directly influences the strength and stiffness characteristics of composites. The brittle phases (e.g., Al_4_C_3_) generated by interfacial reaction or incoherent interface existing in such places has negative effects on mechanical behavior [23,24]. Especially for the nanoscale particles, they bring about an increase in particle–matrix interface concentration higher than that of large particles. Therefore, the structure design of the particle–matrix interface becomes essential. Interestingly, published studies pertaining to NPRAMCs fabricated by in situ methods show clean and reaction-free interfaces [25,26], whereas investigating the relationship between Al matrix structures modified by in situ nanosized particles and tensile behavior is still insufficiently detailed, necessitating a further study.

The Al-Cu-Mn precipitation-hardenable AA2219 alloy has been used for aerospace structures and cryogenic rocket fuel tanks because of its outstanding weldability [27,28,29]. However, limited mechanical properties restricted its further application. To meet the weight-reduction and high-performance demands, efforts have been made by incorporating nanosized particles into the Al-Cu-Mn alloy, resulting in a slightly better performance characteristic [6]. In this paper, AA2219 alloy reinforced by in situ nanosized TiB_2_ particles was first prepared by remelting the master alloys of Al-(Cu, Mn, V, Zr, Ti) and in situ nanosized TiB_2_/Al master composites, following by hot rolling and cold rolling processes. The effect of nanosized TiB_2_ particles on the microstructure evolution (including recrystallization and texture), tensile properties and work-hardening behavior of the present nanocomposite was investigated in detail.

## 2. Experimental Methods

**Figure 1:**
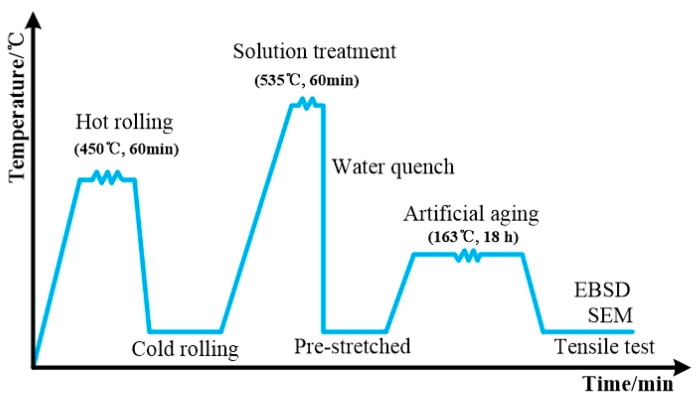
Schematic diagram of sample preparation and testing procedure.

The in situ TiB_2_/AA2219 composite was fabricated with a three-step method. Firstly, an exothermic reaction via mixture salts of K_2_TiF_6_ and KBF_4_ was employed to prepare nanosized TiB_2_/Al matrix composites, and the following reactions occurred: 3K_2_TiF_6_ + 13Al = 3TiAl_3_ + 3KAlF_4_ + K3AlF_6_; 2KBF_4_ + 3Al = AlB_2_ + 2KAlF_4_; AlB_2_ + TiAl_3_ = TiB_2_ + 4Al. Then the intermediate alloys of Al-Cu, Al-Mn, Al-V, Al-Zr, Al-Ti combined with nanosized TiB_2_/Al composites were melted at 760–780 °C and held for 10 min to ensure that the master alloys melted completely. The melt was then vacuum degassed at 740 °C for 10 min after refining, followed by casting at 720–740 °C using metal mold casting to obtain as-cast ingots. Thirdly, the plate with dimensions of 200 mm × 300 mm × 30 mm was machined out from the ingots, and hot rolled to the thickness of 6 mm at 450 °C, homogenized at 530 °C for 24 h, then cold rolled to a thickness of 2 mm at room temperature to obtain the 4 wt.%, 5 wt.% and 6 wt.% nanosized TiB_2_/AA2219 composite sheets. The solution treatment of the present composites sheet was carried out at 535 °C for 1.5 h followed by water quenching, stretched along the rolling direction with the amount of 7%, and artificial aging at 163 °C for 18 h, leading to the T87 temper condition. Schematic diagram of sample preparation and testing procedure was described in Figure 1. For comparison, the AA2219 alloy was prepared and followed by an identical processing and aging history.

The chemical composition (in wt.%) of the matrix alloy and composites was conducted on an inductively coupled plasma atomic emission spectroscopy (ICP-AES, Thermo Fisher Scientific, Waltham, MA, USA) analysis machine, and the results were summarized in Table 1. The phase constitution of the alloy and composites was characterized by a polyfunctional X-ray diffractometer (XRD, D8 ADVANCE Da Vinci, Bruker, Karlsruhe, Germany) with Cu Ka radiation. Tensile tests were performed on a Zwick/Roell machine at strain rate of 1×10^−3^ s^−1^ and each sample was measured at least three times. The transmission electron microscopy (TEM, TALOS F200X, FEI, Hillsboro, OR, USA) and scanning electron microscopy (SEM, Tescan MAIA3, Tescan, Brno, Czech Republic) equipped with Bruker’s e-Flash electron backscatter diffraction (EBSD) detector and Bruker’s XFlash 6|30 energy dispersive spectrometer (EDS)detector was used to characterize the microstructure. Before EBSD characterization, the samples were first mechanically polished with up to 2500 mesh SiC (5 micron) abrasive paper, then fine polished, and finally electropolished by using a hybrid solution of 10% HClO_4_ and 90% ethanol at −20 °C. The Oxford HKL Channel5 software package was used to analyze the EBSD results.

## 3. Results

**Figure 2:**
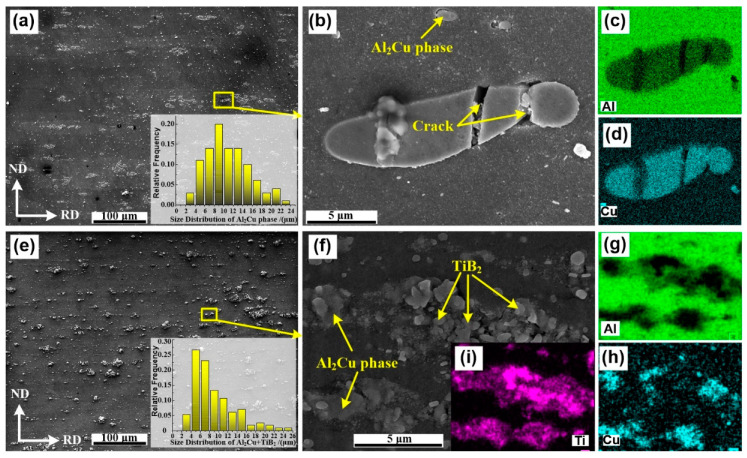
SEM images of (**a**) AA2219 alloy and (**e**) 5 wt.% nanosized TiB_2_/AA2219 composite, (**b**) and (**f**) the morphology of Al_2_Cu phases and the distribution of TiB_2_ nanoparticles, (**c**,**d**) and (**g**–**i**) nergy dispersive spectrometer maps of (**b**) and (**f**).

**Figure 3:**
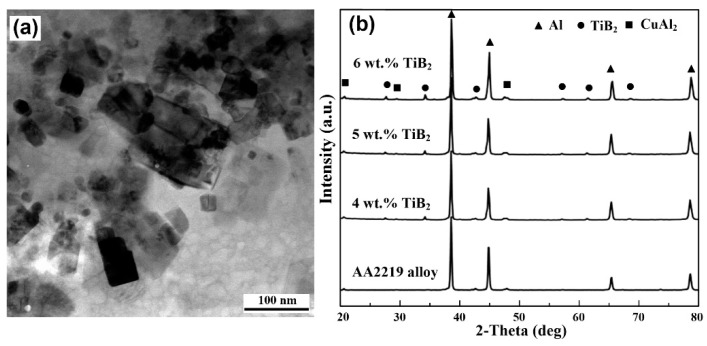
(**a**) TEM image of nanosized TiB_2_ particles, (**b**) XRD pattern for the alloy and composites.

Representative SEM images of AA2219 alloy and the 5 wt.% nanosized TiB_2_/AA2219 composite are shown in Figure 2. Figure 2a reveals a large number of coarse and discontinuous Al_2_Cu phases lying parallel to the rolling direction of the alloy. These Al_2_Cu phases were inhomogeneously distributed and had sizes in the range of 3–20 μm. In this alloy, the concentration of copper (from 5.8 to 6.8 in wt.%) was higher than the limit copper solubility in Al solid solution [30]. It allows precipitation of the maximum number of the secondary phase in the grain interior after aging, while the residual Cu forms these brittle Al_2_Cu phases usually located in grain boundary, as confirmed by XRD characterization in Figure 3b. Once this alloy was subjected to load, these brittle Al_2_Cu phases break first, as shown in Figure 2b. The existed cracks degraded the mechanical properties. Compared with the alloy, the brittle Al_2_Cu phases in the present composite cannot be seen, a hybrid of Al_2_Cu phases and aggregated TiB_2_ nanoparticles was seen instead, as shown in Figure 2c,d as well as in Figure 3a. These hybrids exhibit a size in the range of 3–12 μm. We noticed that the cracks almost vanished in the composite.

**Figure 4:**
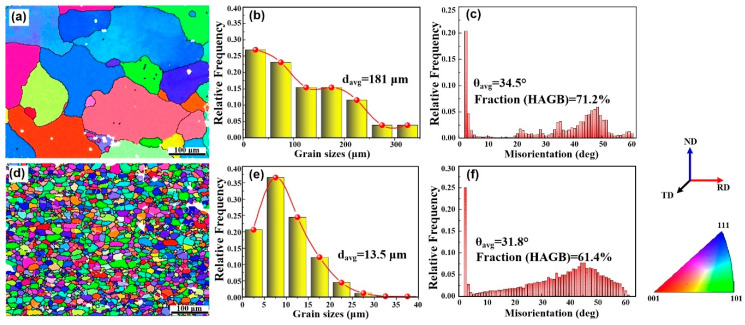
(**a**) and (**d**) inverse pole figure (IPF) maps, (**b**) and (**e**) the distribution of grain sizes, (**c**) and (**f**) misorientation distribution for AA2219 alloy and 5 wt.% nanosized TiB_2_/AA2219 composite.

Figure 4a,d shows inverse pole figure (IPF) maps overlaid with the grain boundary (GB) for AA2219 alloy and the 5 wt.% nanosized TiB_2_/AA2219 composite respectively. The visible white and black lines stand for low-angle grain boundaries (LAGBs, 2–15°) and high-angle grain boundaries (HAGBs, ≥15°). Apparently, the microstructure was drastically affected as nanosized TiB_2_ particles can refine the grains of AA2219 alloy remarkably. The alloy shows an average grain size of ~181 μm, while average grain size of the present composite is ~13.5 μm (see in Figure 4b,e). The distributions of misorientation angles for those present samples can be seen in Figure 4c,f. The result reveals that fraction of HAGBs and average misorientation angle of the alloy are 71.2% and 34.5°, while those for the nanocomposite are 61.4% and 31.8°, respectively.

**Figure 5:**
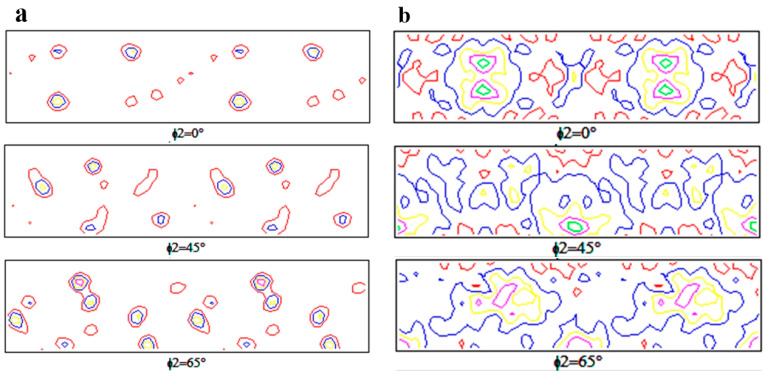
Orientation distribution functions (ODFs) of (**a**) AA2219 alloy and (**b**) 5 wt.% nanosized TiB_2_/AA2219 composite.

**Figure 6:**
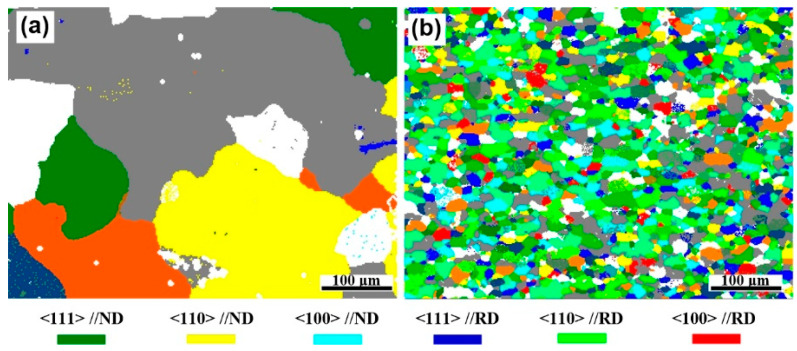
Crystal orientation distribution diagrams of (**a**) AA2219 alloy and (**b**) 5 wt.% nanosized TiB_2_/AA2219 composite.

Studying the evolution of textures in the alloy and the composite is of great significance for studying the correlation between the structure and mechanical properties. The orientation distribution functions (ODFs) with ϕ2 = 0°, 45° and 65° acquired by analyzing the EBSD data from the RD–ND plane of present samples are shown in Figure 5. Generally, the composition of face-centered cubic metal after rolling (Euler angle and Miller index) forms the typical components, such as Brass (110) <112>, Copper (112) <11-1>, S (123)<63-4>. In addition, owing to the recrystallization during the solid solution process, Cube (001)<100> and Goss (110)<001> textures are easily formed [31]. In the present study, Copper, Goss and strong fiber textures of <111>//ND, <110>//ND, <111>//RD can be observed in AA2219 alloy, while the 5 wt.% nanosized TiB_2_/AA2219 composite has fiber textures of <111>//RD, <110>//RD, <100>//RD <111>//ND, <110>//ND, <100>//ND. In order to effectively study texture components, marking typical textures of two samples in Figure 6 and the content fraction of each texture is summarized in Table 2. Different texture contents between the alloy and composites indicates that nanosized particles drastically affect the texture evolution.

**Figure 7:**
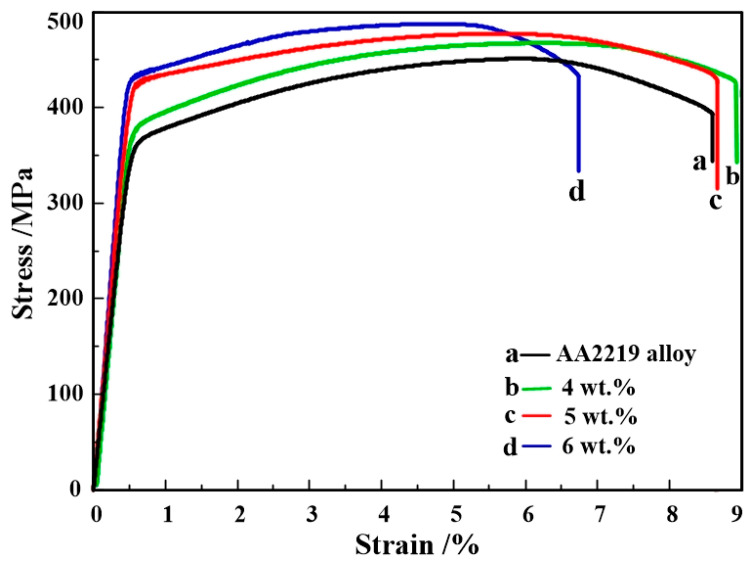
Engineering stress–strain curves of AA2219 alloy and the present composites.

Engineering stress versus strain curves for AA2219 alloy and nanosized TiB_2_/AA2219 composites are described in Figure 7 at room temperature, and their tensile test data and elastic modulus are shown in Table 3. The yield strength (σ_yeild_), ultimate tensile strength (σ_b_), fracture strain (ε_f_), uniform elongation (ε_u_) and elastic modulus of the alloy simultaneously increase when nanosized TiB_2_ particles are added, except for the composites containing 6 wt.% nanosized TiB_2_ particles. the σ_yeild_, σ_b_ and elastic modulus of the 5 wt.% nanosized TiB_2_/AA2219 composite are 11.4%, 5.8% and 5.6% respectively higher than those of the alloy, while its ε_f_ increases slightly. The simultaneously improved tensile properties should be mainly attributed to the changes of the microstructure induced by incorporating nanoparticles. Meanwhile, from the tensile curves, sufficient work-hardening (W-H) rate leads to increase in ε_u_ thus contributing to the enhancement of ε_f_, which will be further discussed later.

## 4. Discussion

From the perspective of dislocations, impeding their motion can achieve high strength [32,33,34], while the spatial distribution, multiplication, and propagation of dislocations are closely related to ductility [35,36]. Accordingly, an approach to improve the strength or ductility invariably requires give-and-take on both sides. However, the present study has exhibited a strength-ductility trade-off, which is closely related to the microstructure modified by nanosized TiB_2_ particles.

**Figure 8:**
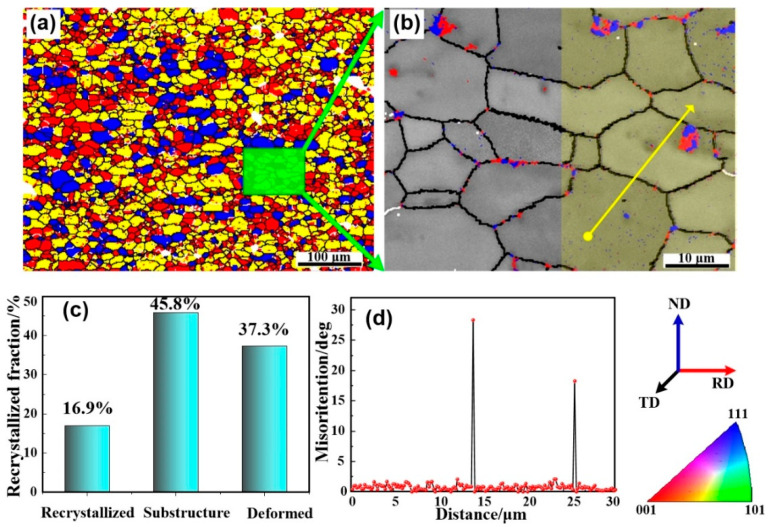
(**a**) Grain boundary (GB) maps overlaid with recrystallized microstructure of the 5 wt.% nanosized TiB_2_/AA2219 composite, in which fully recrystallized grains are in blue, deformed regions in red and substructured grains in yellow, (**b**) Electron backscatter diffraction maps of the composite, the blue and red represent nanoparticles and Al_2_Cu phases, (**c**) recrystallized fraction of the composite, and (**d**) misorientation profiles measured along yellow line.

In AA2219 alloy, the unsolvable Cu usually forms huge brittle Al_2_Cu phases and then distributes around the grain boundary. Whereas those huge Al_2_Cu phases seem to be broken by the aggregated TiB_2_ nanoparticles, as shown in Figure 2. Those small frameworks of Al_2_Cu phases and the aggregated TiB_2_ nanoparticles located at grain boundary will hinder the grain growth during recrystallization (see Figure 4 and Figure 8a). Meanwhile, some of the TiB_2_ nanoparticles dispersed in the grain interior impede recrystallized process, as shown in Figure 8a,b. The grain size of the composite is thus significantly refined. Consequently, fine grain strengthening understood in terms of the Hall–Petch strengthening [37] can explain the enhanced tensile strength.

In addition, textures in the alloy and composite are of great significance to their properties. The composites in this paper exhibit a large amount of fiber textures of <111>//RD, <110>//RD, <100>//RD <111>//ND, <110>//ND, <100>//ND, which is quite different from the alloy. The texture formation mechanism is not the main subject of this paper, which will be discussed in further investigation. Without doubt, the texture evolution is significantly affected by nanosized TiB_2_ particles. Reports indicated that if a small twist angle exists between adjacent grains, cracks can exhibit few if any crack deflections [38]. Thus, those fiber textures in present composite parallel to the ND and RD direction can effectively hinder crack propagation, resulting in enhancement of tensile strength.

From tensile curves shown in Figure 7, the plasticity mechanism also can be explained by work hardening. The Kocks–Mecking model is the basis for studying work hardening [39], in which the average concentration of dislocations (*ρ*) is the main controlling parameter. The Kocks–Mecking model was then extended by Estrin [40,41]. The extended model considered other obstacles (e.g., grain boundaries, second-phase particles) to dislocation motion besides the defects themselves. Hence, when the role of second-phase particles is considered, the evolution of dislocation density with deformation can be expressed by the following equation [40,41]:

(1) $$\frac{d\rho }{d\varepsilon }=K_{d}+K_{1}\sqrt{\rho }-fK_{2}\rho$$

where *K_d_* is the additional dislocations storage on account of the second-phase particles. *K*_1_ represents the dislocation multiplication induced by glide and *K*_2_ stands for the dynamic recovery coefficient, a strain-rate and temperature-dependent parameter, and the recovery rate affected by the second-phase particles is mediated by the factor *f*. Clearly, Equation (1) implies that the second-phase particles increase dislocation accumulation through *K_d_*, and meanwhile slow down dynamic recovery if *f* value < 1. We noticed that the equation mentioned above just suit those second-phase particles which are the nonshearable type [41].

**Figure 9:**
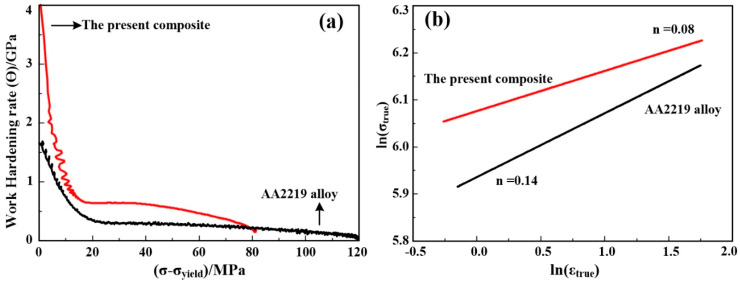
(**a**) Kocks–Mecking plots and (**b**) the ln(σ_true_)-ln(ε_true_) curves for AA2219 alloy and 5 wt.% nanosized TiB_2_/AA2219 composite.

A Kocks–Mecking plot (K–Mp, obtained from the true stress–strain curves) has been demonstrated in Al alloy containing various dislocation obstacles by plotting ϴ (work hardening rate) = *d*σ/*d*ε against (σ − σ_yield_) [42,43]. Among the obtained parameters, Y-intercept (ϴ_max_) represented the initial W-H rate, and the slope (−*d*ϴ/*d*(σ − σ_yield_)) is proportional to the dynamic recovery rate [41]. The results of the K-Mps for the alloy and 5 wt.% nanosized TiB_2_/AA2219 composite are shown in Figure 9a, and calculated W-H behaviors are given in Table 4. Clearly, the ϴ_max_ (expressed by the term *K_d_* of Equation (1)) value for the alloy is 1650 MPa, while that for the composite is 4394 MPa. Kocks and Mecking have been pointed out that the ϴ_max_ ~G/20 (G is the shear modulus and the value for Al is 26,000 MPa) in a well-annealed FCC metals [39]. So, the difference (1650MPa-/20MPa = 350MPa) for the alloy can be contributed to the precipitate phases of θ″ or θ′. As for the dynamic recovery rate, nanosized TiB_2_ particles sharply reduced that phenomenon because of the decrease in −*d*ϴ/*d*(σ − σ_yield_), which can be observed in Table 4.

The analysis above clearly shows that nanosized TiB_2_ particles enhance the W-H rate. This can be concluded by observing the shape of the stress–strain curves and the numerical values of the Hollomon equation exponent (*n*, see Figure 9b and Table 4) as well as the difference between maximum and yield strength (σ_b_ − σ_yield_). It can be inferred from Equation (1) that nanosized TiB_2_ particles increase the parameter *K_d_* (dislocation accumulation owing to nanoparticles) and decrease the *K*_2_ (dynamic recovery rate due to dislocation pinning by nanoparticles). Hence, the increase in W-H rate was attributed to dislocation generation/storage and the concurrent decrease of the dynamic recovery rate.

**Figure 10:**
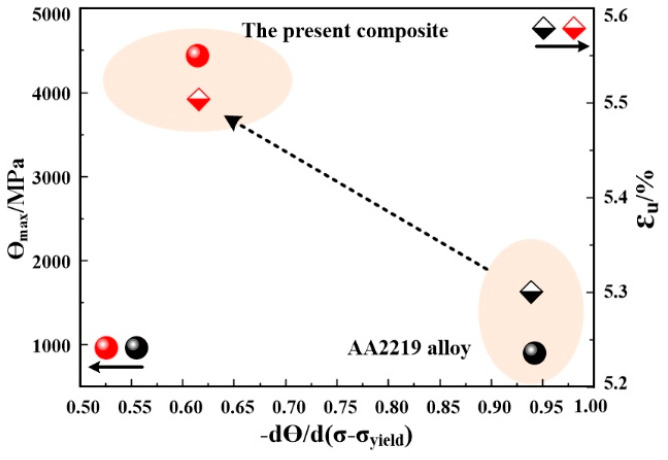
Correlation between uniform elongation, initial work-hardening (W-H) rate and dynamic recovery rate.

The practical implications of this work can be summarized as a plot of uniform elongation and initial work-hardening rate against dynamic recovery rate in Figure 10. The initial work-hardening rate and dynamic recovery rate of the present composite are represented by the red sphere and quadrilateral, while those for AA2219 alloy are represented by the black sphere and quadrilateral. It is apparent that an inverse relationship holds between NPRMACs and its matrix alloy. Therefore, in order to produce new structural materials with high strength while maintaining respectable ductility as well as excellence forming behavior, the addition of a small well dispersion nanoparticles is a valid strategy.

## 5. Conclusions

In comparison with AA2219 alloy, the yield strength, tensile strength and elastic modulus of the present composite simultaneously increased, while its fracture strain almost remained unchanged. When 5 wt.% nanosized TiB_2_ particles were added, the yield strength, tensile strength and elastic modulus increased by 11.4%, 5.8% and 5.6%, respectively. The enhanced strength and ductility exhibited a correlation with the microstructure modified by nanosized TiB_2_ particles. For the first, the recrystallization of the matrix alloy was impeded by nanosized TiB_2_ particles, and, meanwhile, the hybrids of nanosized TiB_2_ particles and Al_2_Cu phases located at the grain boundary hindered the grain growth during recrystallization, leading to grain refinement. Secondly, a large amount of fiber textures of <111>//RD, <110>//RD, <100>//RD <111>//ND, <110>//ND and <100>//ND formed in the present composite hinder crack propagation. Thirdly, nanosized TiB_2_ particles enhanced W-H rate by raising the dislocation generation/storage and meanwhile decreasing the dynamic recovery rate, resulting in an increase in uniform elongation. Our findings provide a practical method to improve simultaneously strength and plasticity of particle-reinforced Al matrix composites.

## Figures and Tables

**Table 1:** The mass fractions of chemical compositions of the alloy and composite.

Samples	Cu	Mn	V	Zr	Ti	B	Al
AA2219	6.31	0.27	0.06	0.13	0.03	-	Bal.
4 wt.%	6.21	0.28	0.09	0.12	2.89	1.19	Bal.
5 wt.%	6.37	0.31	0.09	0.12	3.51	1.54	Bal.
6 wt.%	6.28	0.31	0.09	0.11	4.12	1.87	Bal.

**Table 2:** Textures content of AA2219 alloy and 5 wt.% nanosized TiB_2_/AA2219 composite.

Samples	Copper	Goss	<111>//RD	<110>//RD	<100>//RD	<111>//ND	<110>//ND	<100>//ND
alloy	13.9%	10.7%	26.4%	-	-	12.1%	45.5%	-
composite	-	-	20.1%	32%	11.1%	13%	26.1%	11.7%

**Table 3:** Tensile test data and elastic modulus of AA2219 alloy and the composite.

Samples	σ_yeild_/MPa	σ_b_/MPa	ε_f_/%	ε_u_/%	Elastic Modulus/GPa
AA2219	368 ± 7	451 ± 6	8.6 ± 0.6	5.3 ± 0.5	72.8
4 wt.%	382 ± 5	460 ± 7	8.9 ± 0.8	5.8 ± 0.6	75.1
5 wt.%	410 ± 10	477 ± 5	8.7 ± 0.9	5.5 ± 0.3	76.9
6 wt.%	414 ± 5	489 ± 10	6.7 ± 1.0	4.6 ± 0.8	78.6
Al-6Cu [26]	345	451	11.0	-	-
NdB_6_/Al-5.0Cu [6]	298	516	8.3	-	-

**Table 4:** Hollomon equation exponent (*n*) values and work-hardening parameters of AA2219 alloy and 5 wt.% nanosized TiB_2_/AA2219 composite.

Samples	ϴ_max_	−*d*ϴ/*d*(σ − σ_yield_)	*n*
AA2219	1650	0.94	0.14
The composite	4394	0.62	0.08
